# Identification of Suitable Drug Combinations for Treating COVID-19 Using a Novel Machine Learning Approach: The RAIN Method

**DOI:** 10.3390/life12091456

**Published:** 2022-09-19

**Authors:** Aliakbar Kiaei, Nader Salari, Mahnaz Boush, Kamran Mansouri, Amin Hosseinian-Far, Hooman Ghasemi, Masoud Mohammadi

**Affiliations:** 1 Department of Computer Engineering, Sharif University of Technology, Tehran 1136511155, Iran; 2 Department of Biostatistics, School of Health, Kermanshah University of Medical Sciences, Kermanshah 6715847141, Iran; 3 Department of Industrial Engineering, Shahid Beheshti University of Medical Sciences, Tehran 1968917313, Iran; 4 Medical Biology Research Centre, Kermanshah University of Medical Sciences, Kermanshah 6714415185, Iran; 5 Department of Business Systems and Operations, University of Northampton, Northampton NN1 5PH, UK; 6 Student Research Committee, Kermanshah University of Medical Sciences, Kermanshah 6734667149, Iran; 7 Cellular and Molecular Research Center, Gerash University of Medical Sciences, Gerash 7441758666, Iran

**Keywords:** RAIN method, machine learning, COVID-19, treatment of patients, drugs combinations, network meta-analysis

## Abstract

**Simple Summary:**

This study follows an improved approach to systematic reviews, called the Systematic Review and Artificial Intelligence Network Meta-Analysis (RAIN), registered within PROSPERO (CRD42021256797), in which, the PRISMA criterion is still considered. Drugs used in the treatment of COVID-19 were searched in the databases of ScienceDirect, Web of Science (WoS), ProQuest, Embase, Medline (PubMed), and Scopus. In addition, using artificial intelligence and the measurement of the *p*-value between human genes affected by COVID-19 and drugs that have been suggested by clinical experts, and reported within the identified research papers, suitable drug combinations are proposed for the treatment of COVID-19. During the systematic review process, 39 studies were selected. Our analysis shows that most of the reported drugs, such as azithromycin and hydroxyl-chloroquine on their own, do not have much of an effect on the recovery of COVID-19 patients. Based on the result of the new artificial intelligence, on the other hand, at a significance level of less than 0.05, the combination of the two drugs therapeutic corticosteroid + camostat with a significance level of 0.02, remdesivir + azithromycin with a significance level of 0.03, and interleukin 1 receptor antagonist protein + camostat with a significance level 0.02 are considered far more effective for the treatment of COVID-19 and are therefore recommended.

**Abstract:**

COVID-19 affects several human genes, each with its own *p*-value. The combination of drugs associated with these genes with small *p*-values may lead to an estimation of the combined *p*-value between COVID-19 and some drug combinations, thereby increasing the effectiveness of these combinations in defeating the disease. Based on human genes, we introduced a new machine learning method that offers an effective drug combination with low combined *p*-values between them and COVID-19. This study follows an improved approach to systematic reviews, called the Systematic Review and Artificial Intelligence Network Meta-Analysis (RAIN), registered within PROSPERO (CRD42021256797), in which, the PRISMA criterion is still considered. Drugs used in the treatment of COVID-19 were searched in the databases of ScienceDirect, Web of Science (WoS), ProQuest, Embase, Medline (PubMed), and Scopus. In addition, using artificial intelligence and the measurement of the *p*-value between human genes affected by COVID-19 and drugs that have been suggested by clinical experts, and reported within the identified research papers, suitable drug combinations are proposed for the treatment of COVID-19. During the systematic review process, 39 studies were selected. Our analysis shows that most of the reported drugs, such as azithromycin and hydroxyl-chloroquine on their own, do not have much of an effect on the recovery of COVID-19 patients. Based on the result of the new artificial intelligence, on the other hand, at a significance level of less than 0.05, the combination of the two drugs therapeutic corticosteroid + camostat with a significance level of 0.02, remdesivir + azithromycin with a significance level of 0.03, and interleukin 1 receptor antagonist protein + camostat with a significance level 0.02 are considered far more effective for the treatment of COVID-19 and are therefore recommended. Additionally, at a significance level of less than 0.01, the combination of interleukin 1 receptor antagonist protein + camostat + azithromycin + tocilizumab + oseltamivir with a significance level of 0.006, and the combination of interleukin 1 receptor antagonist protein + camostat + chloroquine + favipiravir + tocilizumab7 with corticosteroid + camostat + oseltamivir + remdesivir + tocilizumab at a significant level of 0.009 are effective in the treatment of patients with COVID-19 and are also recommended. The results of this study provide sets of effective drug combinations for the treatment of patients with COVID-19. In addition, the new artificial intelligence used in the RAIN method could provide a forward-looking approach to clinical trial studies, which could also be used effectively in the treatment of diseases such as cancer.

## 1. Introduction

Coronavirus 2019 (COVID-19) is an infectious disease caused by severe acute respiratory coronavirus (SARS-CoV-2). The disease was first identified in December 2019 in Wuhan, the capital of China’s Hubei Province, and has since spread worldwide [1]. The virus is mainly transmitted between people during close contact, often through small droplets produced by coughing, sneezing, or talking [2].

Today, the international community and healthcare professionals are affected by the epidemic of this coronavirus (COVID-19) [3]. Although most patients with this disease have mild infectious symptoms, about 20% of patients develop severe and acute pneumonia, which can lead to death [4]. In addition to respiratory symptoms, COVID-19 causes widespread inflammation and severe damage to other organs. The activation of immune cells, release of inflammatory factors such as interleukin and tumor necrosis factor-alpha, D-dimer, ferritin, and C-reactive protein, and activation of platelets cause inflammation and widespread vascular disorders due to this disease [5]. Damages to the heart [6], brain [7], kidney [8], and liver [9] are also observed due to severe manifestations of the disease.

Standard supportive care, corticosteroids, intravenous immunoglobulin, and experimental or alternative antiviral therapies (such as ramsedivir, ribavirin, lupinavir-ritonavir, amifenovir, and interferons, etc.) have been different treatment strategies for patients with COVID-19 [4]. To date, none of the proposed therapies have been introduced as the main treatment for this disease. However, in the face of the pressures caused by the pandemic, health care workers around the world were forced to prescribe these drugs despite inconclusive clinical evidence [10].

An effective treatment strategy worldwide is essential for the rapid treatment of patients with COVID-19. There are a huge amount of COVID-19 treatment data in healthcare clinics around the world, which demonstrates the need for advanced methods of analysis well [11]. Artificial intelligence is one of the methods that can easily track the prevalence of COVID-19, identify at-risk patients, and be useful in controlling this infection in real time [12]. Artificial intelligence techniques also make it possible to analyze patients’ previous data, predict the risk of death, and help fight the virus with population screening, medical assistance, information, and infection control suggestions. This technology, as an evidence-based medical tool, can improve the planning, treatment, and reported outcomes of COVID-19 patients [13,14]

Network meta-analysis is another way to find effective drugs for the treatment of a disease. Using network meta-analyses based on existing clinical trials, high-quality comparative evidence can be generated to evaluate the drugs used to treat COVID-19 [15]. In addition, a network meta-analysis model can estimate treatment effects even for treatments that have never been directly compared in a direct study [16].

Systematic review and meta-analysis articles have been widely used by researchers worldwide, especially in the COVID-19 pandemic. The international community and researchers have always tried to counteract COVID-19 by determining the target group of the disease, methods of prevention, diagnosis, treatment, and vaccine discovery, which have also had noteworthy effects. However, due to the wide range of emerging COVID-19 variations due to mutations, despite the discovery of a number of vaccines, we still have several patients who require specialist medical care. We still do not know: what drug combinations and what diets can be useful for treating patients; whether Chinese medicine and common medical treatments can be used in the treatment of this disease or not; and whether children manifest the same symptoms as adults. Therefore, researchers globally should seriously consider the need for certainty and validity of information in order to save patients’ lives. In this study, we assessed the impact and application of artificial intelligence to provide responses to some of the above questions by conducting a novel systematic review and meta-analysis. This novel systematic review approach is referred to as Systematic Review and Artificial Intelligence Network Meta-Analysis (RAIN), and the research protocol was registered within the PROSPERO international prospective register of systematic reviews, (CRD42021256797).

## 2. Materials and Methods

This new protocol has three general stages, each of which entails a number of activities. The first stage includes a systematic review that entails the review, the results of the identified and selected studies, and summaries of the clinical results. The second stage includes a meta-analysis of the results, if necessary, to provide quantitative information (this section can be skipped if the researchers find that the heterogeneity among the selected studies is high and that meta-analysis is not possible). Finally, the third stage is the analysis of the results of one-on-one studies using artificial intelligence and a machine learning approach. In this stage, artificial intelligence proposes an approach using specific scenarios at the significance level of 0.05 and 0.01 by reviewing the results of one-on-one studies and adding and subtracting results and variables. The study outlined in this manuscript followed the same set of phases of RAIN:

### 2.1. Stage I: Systematic Review

The purpose of the systematic review is to collect all of the information obtained from studies conducted on the treatments for COVID-19 patients and to extract the reported results within the selected studies. The systematic review section was performed in accordance with the Cochrane 7-step approach, including: research question selection, defining inclusion and exclusion criteria, article identification, study selection, study quality evaluation, data extraction, and analysis and interpretation of findings.

### 2.2. Research Question and Keyword Determination according to PICO Instructions

According to the PICO guidelines, the research question is: “What are the most effective drugs and drug combinations for the treatment of patients with COVID-19?”. The study population included patients with COVID-19 worldwide, the intervention included therapeutic measures and the use of specific drugs for the treatment of COVID-19 patients, comparison included an evaluation of the effect of specific drugs in the study with different drug therapies, and the desired outcome was explained in accordance with the outcome including the effect or not of the drug in the treatment of the disease.

### 2.3. Searching Articles

The keywords were extracted from the Medical Subject Headings (MeSH) browser and in accordance with the PICO instructions. For each question, keywords were selected separately and in relation to the study population (P), intervention (I), comparison (C), and outcome (O).

### 2.4. Identifying Articles

Initially, a large number of abstracts of articles in which COVID-19 was mentioned were discovered by two researchers in our study. Through text mining, keywords related to COVID-19 were automatically ranked (e.g., affected human genes, effective drugs, etc.) to increase the accuracy of the results obtained by the researchers, and not to miss a related keyword.

International databases of ScienceDirect, Web of Science (WoS), ProQuest, Embase, Medline (PubMed), and Scopus were searched with lower time limit of 1 December 2019, and up to 31 December 2021. Articles published in English as well as articles in other languages, yet with an abstract in English, were reviewed. The search strategy for each database was determined through the Advance Search, and was followed using all possible keyword combinations and using AND and OR operators.

### 2.5. Study Selections, Based on Inclusion and Exclusion Criteria

The reference lists of all articles that met the inclusion criteria were manually reviewed to access further relevant studies. To avoid errors, mistakes, and also bias, all steps of article search, study selection, quality evaluation, and data extraction were performed by two reviewers (researchers) independently. If there was a difference in opinion among the reviewers regarding the inclusion of an article in our study, a third reviewer reviewed the article. The search process was also reconstructed by artificial intelligence and the machine learning process to increase search accuracy.

The information of all of the articles found in each database was transferred into the EndNote X8 reference management software and then in Python in the form of a data frame. After searching all of the databases, duplicate articles were removed. Then, in order to avoid the risk of bias in the selection of studies, the names of the authors and the titles of the journals were omitted and a checklist was prepared based on the titles and abstracts of the studies. Studies where their full text was not found and where they did not meet the inclusion criteria were excluded from the systematic review process. The full texts of all remaining articles were then evaluated [10,11].

### 2.6. Inclusion and Exclusion Criteria according to the Research Question

Studies involving specific drugs or drug combinations for the treatment of patients with COVID-19 globally, including clinical trial studies, were included in our work. Observational research, including case studies, cohorts, descriptive studies, rare case reports, review studies, and meta-analyses, were all excluded.

### 2.7. Quality Evaluation of Studies

In order to evaluate the quality of the studies, the CONSORT checklist was adopted. This checklist is helpful in reviewing clinical trial studies, and includes 25 general items, each with sub-items (a total of 37 sub-items). The items of this checklist include: Title, Abstract, Background, Objectives, Methods, Participants, Interventions, Objectives, Consequences, Sample Size, Randomization, Participants flow, Blinding, etc. In order to rate the articles, if each article referred to the items considered within the checklist, it was given a score of 1 and, if it was not mentioned, a score of zero was allocated. The minimum and maximum scores in this checklist are 0 and 37, respectively. Studies with 75% or more of the maximum achievable score (score greater than or equal to 27) were considered as “high quality”, studies with a score between 50–75% (score 18–26) were considered as “average quality”, and studies with a score lower than 50% (score less than or equal to 17) were considered as “low quality” studies [12].

### 2.8. Data Extraction

**Figure 1:**
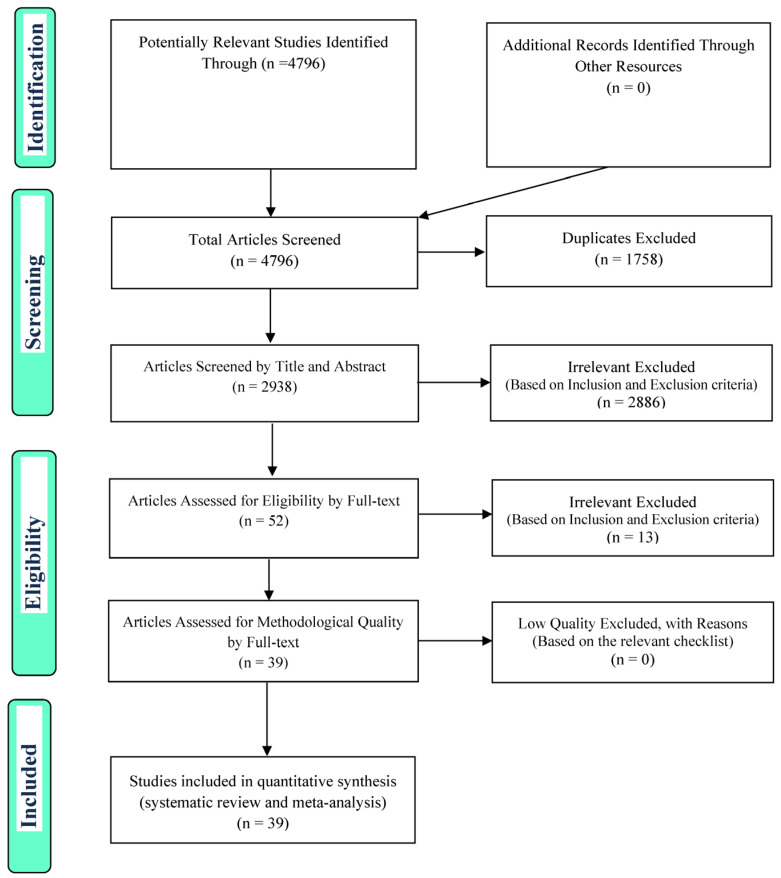
The flowchart on the stages of including the studies in the systematic review and meta-analysis (PRISMA 2009).

After selecting the studies in the systematic review, in order to select the studies to enter the meta-analysis, the four steps of Preferred Reporting Items for Systematic Reviews and Meta-Analyses (PRISMA) 2009 process were considered, i.e., article identification, screening, eligibility assessment, and, finally, inclusion of articles that can also be submitted for meta-analysis (Figure 1).

### 2.9. Stage 2: Artificial Intelligence

At this stage, the results of studies obtained from the network meta-analysis were imported to be used by AI algorithm. The AI algorithm receives the effectiveness of each drug in the affected human genes by COVID-19 as input, and, in the output, provides some effective scenarios. Each scenario includes the drug combinations proposed for the treatment of COVID-19.

The entire genome sequence of COVID-19 was constructed by researchers, and the following proteins are some of the identified human genes that are mostly affected by COVID-19: ‘ACE2’, ‘TMPRSS2’, ‘CDSN’, ‘CRP’, ‘IL6’, ‘FURIN’, ‘SH2D3C’, ‘IGHV3-53’, ‘LOC100506985’, ‘SNORA81’, ‘PORCN’, ‘F2’, ‘IL1RN’, ‘IL6R’, ‘RGAG4’, ‘SH2D3A’, ‘SLC6A19’, ‘DPP4’, ‘SNORD35B’, ‘TMPRSS11B’, ‘BSG’, ‘TP53’, ‘ZCCHC16’, ‘LDOC1L’, ‘ACE’, ’IFITM3’, ‘COX18’, ‘RGAG1’, ‘CENPJ’, ‘FUT3’, ‘VTN’, ‘WASH1’, ‘BMND8’, ‘BMND7’, ‘BCL2’, ‘DNAH7’, ‘PSMD1’, ‘CAT’, ‘NAA50’, ‘TMEM189-UBE2V1’, ‘TRBV11-2’, ‘IL1F10’.

From the viewpoint of genome sequencing, COVID-19 is 79% similar to SARS-CoV, 51.8% similar to MERS-CoV, and around 87.6% similar to other SAR-like CoVs of Chinese horseshoe bats (called ZC45 and ZXC21).

These results suggest that the virus originated from bats [17,18]. A possible agent is a SARS-CoV-2 coronavirus of the genus BETACORONAVIRUS, which is a genus of the CORONAVIRIDAE family, which mainly causes respiratory or gastrointestinal disorders in a large number of mammals [19]. Human beta coronaviruses include HUMAN ENTERIC CORONAVIRUS, HUMAN CORONAVIRUS OC43, MERS VIRUS, and SARS VIRUS [20]. Members have central regulatory 5′-CUAAAC-3′ or 5′-CUAAAC-3′ transcription sequences and most do not have ORF downstream to the N protein gene1. From the other point of view, in order to find the similarity between proteins of COVID-19 and nucleotides, basic local alignment search tool (BLAST) is one of the online solutions.

The percent identity, E-value, and query cover measures of the National Center for Biotechnology Information (NCBI) find the most similar nucleotides with these proteins. The first measure is to find how similar the query sequence is to the associated nucleotides. The second one describes how many times it would be expected to match by chance in a database of that size. The third one describes how much of the query sequence is covered by the target sequence. To carry this out, we found the most similar nucleotides related to each protein, separately. Then, the results of each measure were aggregated. Table 1 shows the mean and standard deviation for each measure. The first column shows nucleotides that are the most similar to the COVID-19 proteins (Table 1).

### 2.10. Combining p-Values

**Figure 2:**
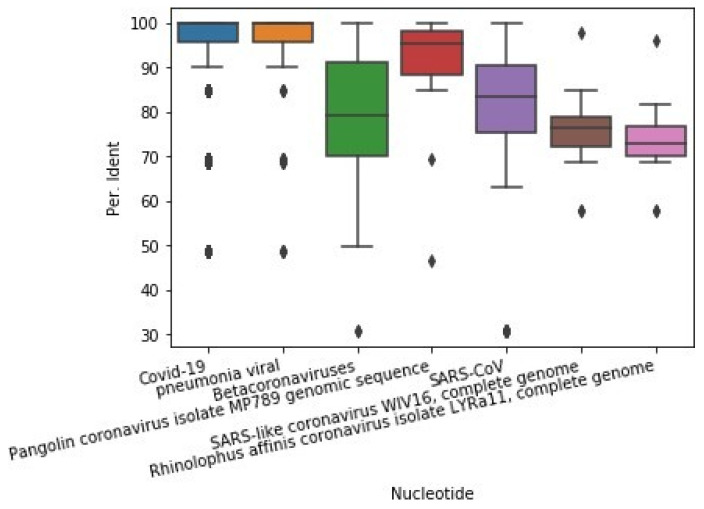
Boxplot of percent identity between COVID-19 proteins and similar nucleotides.

Evolutionary biologists have long used meta-analytic tactics to combine evidence from several existing studies. If raw data cannot be summarized, *p*-value-based meta-analysis offers a hands-on method that can be practically as influential as combining data. Many common *p*-value combining methods have the same general shape. At first, the *p*-value for the *i*-th study, *p_i_*, is transformed by a function H. Then, in the new distribution, one type of mean, such as arithmetic or weighted ones, is used to calculate the combination $\;T=\sum w_{i}H\left(p_{i}\right).$ Finally, the inverse transform of *T* is computed as the result of the combined *p*-value. Two common methods used to combine *p*-values are Fisher’s methods and Stouffer’s method. Fisher’s method combines *p*-values into one test statistic (chi-squared). The boxplot of Table 1 when considering the present identity is shown in Figure 2.

### 2.11. Using the Formula


$$X_{2k}^{2}∼-2\overset{k}{\underset{i=1}{\sum }}\ln\left(p_{i}\right)$$

In the interval [0,1], the negative logarithm of this distribution reveals a quantity that follows exponential distribution. Moreover, when scaling a value that follows an exponential one by a factor of two, it will follow a chi-squared distribution with two degrees of freedom. Fisher’s method computes the sum of these *k*-independent chi-squared values, where it follows a chi-squared distribution with 2*k* degrees of freedom. However, this method does not use any weighting approach, whereas, for our aim, the sizes of studies are important.

Stouffer’s method, as another known work, uses the inverse normal distribution function to transform the uniform distribution, allowing for the incorporation of study weights using z-scores [21]. One of its improvements is Lipták’s method, in which it considers weights in the Stouffer’s method [22]. This method is also known as the weighted Z-test and the combined *p*-value is computed by:

$$P_{z}=1-\emptyset \frac{\sum_{i=1}^{k}w_{i}z_{i}}{\sqrt{\sum_{i=1}^{k}w_{i}^{2}}}$$

where *i* is the number of study from *k* studies, *p_i_* is its *p*-value, *w_i_* is its weight, *Z_i_* = ∅^−1^ (1 − *p_i_*), and ∅ indicates the standard normal cumulative distribution function [23]. Taking into account the correct weights leads to a better combination. If only study sample sizes are available, their square roots converge for an optimal result [22]. Won et al. demonstrated that the effect size over the standard error has the optimal power [24]. We used a combination method based on the weighted version of power mean in the transformed distribution. We proposed a machine learning method that uses *p*-values between the COVID-19 and affected human genes, as well as *p*-values between those genes and drug associations as input. The output finds associations’ cooperations (drug recipe in this article) that make small combined *p*-value with COVID-19.

### 2.12. Power Means

To combine sets of numbers, power means are a type of function that implements positive real numbers. The power means of *p*_1_,…, *p_n_* are calculated by [25]:

$$M_{k}\left(p_{1},\ldots ,\;p_{n}\right)={\left(\frac{1}{n}\overset{n}{\underset{i=1}{\sum }}p_{i}^{k}\right)}^{\frac{1}{k}}$$

*M*_−__∞_ is the minimum function, *M*_−1_ is the harmonic mean, *M*_0_ is the geometric mean, *M*_1_ is the arithmetic mean, *M*_2_ is the quadratic mean, *M*_3_ is the cubic mean, and *M*_−__∞_ is the maximum of *p*_1_,…, *p_n_* [26]. With *k* = 0, the power means summarize to the geometric mean:

$$limM_{\to 0}\left(p_{1},\ldots ,\;p_{n}\right)={\left(\overset{n}{\underset{i=1}{\prod }}p_{i}\right)}^{\frac{1}{n}}$$

Considering that each *p_i_* has the weight *w_i_*, the weighted geometric mean is computed by:

$$P={\left(\overset{n}{\underset{i=1}{\prod }}p_{i}^{w_{i}}\right)}^{\frac{1}{\sum_{i=1}^{n}w_{i}}}$$

### 2.13. The Proposed AI Models

**Figure 3:**
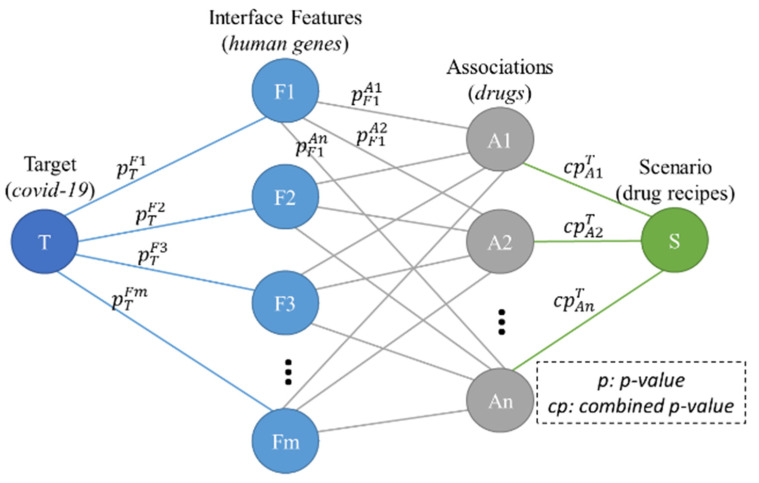
Structure of proposed method (p-NA).

The proposed p-nearest associations algorithm (p-NA) is a nonparametric method used to propose associations’ cooperations for the target. The input consists of associations where the *p*-values between them and interface features are known from the network meta-analysis. The output contains associations’ cooperations that have a small significance. Figure 3 shows the overall structure of p-NA.

The proposed model consists of forward and backward steps. In the forward step, the proposed p-NA algorithm computes all combined *p*-values between associations (drugs) and the target (COVID-19). Then, the association with the smallest combined *p*-value is selected. The combined *p*-value is calculated based on interface features (human genes). In the backward step, based on the selected association(s), all *p*-values between interface features and the target are updated. The significance of cooperating associations in each scenario is computed by multiplying the combined *p*-values of those associations in that scenario. These steps are iterated until that significance becomes less than a threshold (Figure 3).

### 2.14. The Forward Step

**Figure 4:**
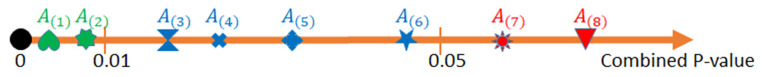
Selecting association with the smallest combined *p*-value with the target.

Suppose we have pairs $\left(A1,cp_{A1}^{T}\right),\ldots ,\;\left(An,cp_{An}^{T}\right)$, where $c_{Ai}^{T}$ is the combined *p*-value between the ith association and the target. Let $\left(A_{\left(1\right)},\;cp_{1}\right),\ldots ,\;\left(A_{\left(n\right)},\;cp_{n}\right)$ be a reordering of the training data such that $cp_{\left(1\right)}\leq \ldots \leq cp_{n}$. Figure 4 shows an example of the p-NA algorithm. The black circle on the left is the target and the associations are embedded in the axis with their combined *p*-values with the target. The associations with combined *p*-values less than 0.01 are shown in green $\left(i.e.,\;A_{\left(1\right)},A_{\left(2\right)}\right)$ and the others that are less than *p* = 0.05 are blue $\left(i.e.,A_{\left(3\right)},A_{\left(6\right)}\right)$. Other ones are shown in red (Figure 4).

In other words, if the combined *p*-values between associations and the target are found, the forward step simply chooses the nearest association with the viewpoint of combined *p*-values. Thus, the forward step is summarized as computing the combined *p*-values between associations and the target.

Under the null hypothesis, the *p*-values follow the uniform distribution on the interval [0,1]. The natural logarithm of a uniformly distributed value follows an exponential distribution. The weighted mean of that exponential distribution is $\frac{\sum_{i}^{n}w_{i}lnp_{i}}{\sum_{i=1}^{n}w_{i}}$, where $p_{i}$ is the *p*-value for $p^{i}$ association. Finally, the inverse transform of the logarithm yields a quantity that follows a uniform distribution on the interval [0,1]. It is the same as the weighted geometric mean (WGM) in the original distribution. The following formula shows the combined *p*-value between an association and the target:

$$cp_{Aj}^{T}=\exp\left(\frac{\sum_{i=1}^{m}w_{T}^{Fi}\ln\left(P_{Fi}^{Aj}\right)}{\sum_{i=1}^{m}w_{T}^{Fi}}\right)=\left(\overset{m}{\underset{i=1}{\prod }}({\left(p_{Fi}^{Aj}\right)}^{w_{T}^{Fi}}\right){)}^{\frac{1}{\sum_{i=1}^{m}w_{T}^{Fi}}}$$

where $cp_{Aj}^{T}$ is the combined *p*-value between the *j*-th association and the target, $w_{T}^{Fi}=1-p_{T}^{Fi}$ is the weight between the *i*-th interface feature and the target, $P_{T}^{Fi}$ is the *p*-value between the target and *i*-th interface feature, and $P_{Fi}^{Aj}$ is the *p*-value between the i-th interface feature and *j*-th association.

As a simple case of a genome with just two genes (interface features), Figure 3 shows the construction of a combined *p*-value for just one association. Assume that two genes are independent and thus perpendicular to each other.

**Figure 5:**
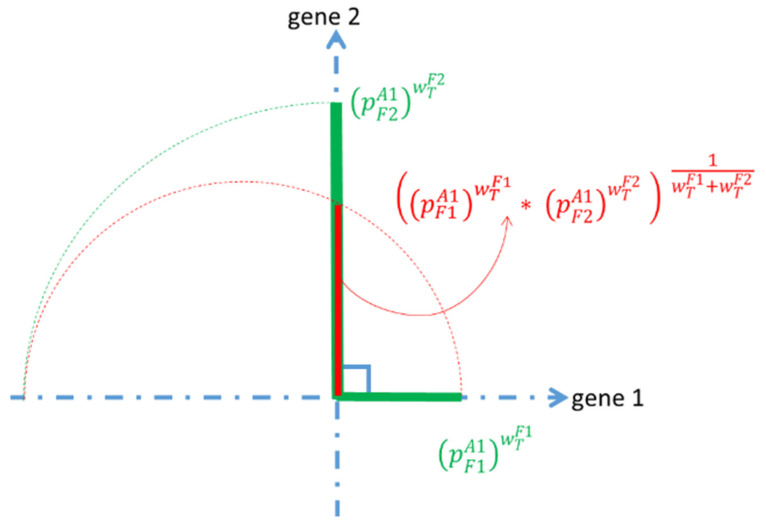
Measure of combined *p*-value.

$p_{F1}^{A1},p_{F2}^{A1}$ is the *p*-value between these genes and the association. The weight of gene i is $w_{T}^{Fi}$. Rotating the line segment ${\left(p_{F2}^{A1}\right)}^{w_{T}^{F2}}$ in the direction of gene 1 is shown with a dashed green curve. A circle with its center in the gene 1 line that touches the ${\left(p_{F1}^{A1}\right)}^{w_{T}^{F1}}$ and rotated line segment of ${\left(p_{F2}^{A1}\right)}^{w_{T}^{F2}}$ is shown in a red dashed curve. The measure of the combined *p*-value is shown red line (Figure 5).

Therefore, the combined *p*-values between associations and the target are computed and the association with the smallest combined *p*-value is selected as the p-nearest association.

For this paper’s case study, i.e., the drugs recipe for COVID-19, the *p*-values between drugs and affected human genes, and *p*-values between affected human genes and COVID-19, are available at Coremine Medical online database. It uses text mining algorithms to search and explore biomedical connections.

**Figure 6:**
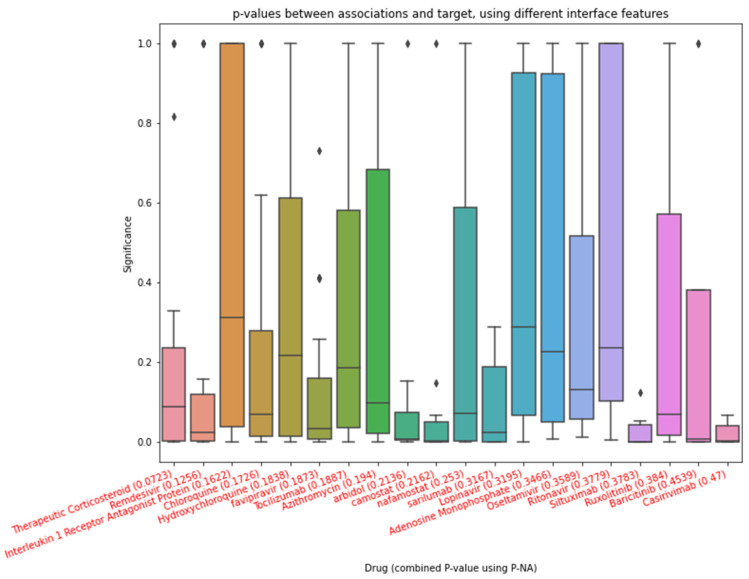
The 20 drugs with the smallest combined *p*-values between them and COVID-19 in parentheses.

After obtaining the drugs suggested by clinical experts, the human genes affected by COVID-19, we collected all of the *p*-values between drugs and genes and between COVID-19 and genes. We separated each association and then found the combined *p*-value using the proposed WGM. The nearest association was then found in the forward step of p-NA. Figure 6 shows the results of p-NA’s forward step for associations in the drug categories.

We also used the proposed p-NA to find nearest associations in categories such as foods, MeSH, molecular function, gene/protein, or biological process 3. However, this paper focuses on drugs 4.

### 2.15. The Backward Step

In this step, the proposed p-NA algorithm updates the weights between the target and all interface features. This is computed by multiplying the weights with the *p*-values of the selected association and those interface features; i.e., (Figure 6):

$$w_{T}^{Fi}=w_{T}^{Fi}\times p_{Fi}^{A_{selected}}$$

where $A_{selected}$ is the selected association in the forward step. Obviously, $p_{T}^{Fi}$ is updated by $1-w_{T}^{Fi}$.

## 3. Results

### 3.1. Systematic Review

The articles used in this study were published in the period 2020-2021. Of the 39 confirmed articles, most studies were conducted in the United States [27,28,29,30,31,32,33,34,35,36,37], as well as 8 other studies in Iran [38,39,40,41,42,43,44,45], 4 studies in Spain [46,47,48,49], 3 studies in France [50,51,52], 2 studies in the UK [53,54] and Brazil [55,56], and 1 study in each of Norway [57], China [58], Switzerland [59], Italy [60], Canada [61], Egypt [62], Taiwan [63], and Greece [64]. The highest study population was observed in the study of Horby et al. [54] with 5030 participants.

Most studies on patients with COVID-19 evaluated the effect of HCQ [27,28,29,30,34,35,36,47,49,50,51,53,55,57,58,61,62,63]. In two interventional studies without the presence of the control group, the effect of HCQ on reducing mortality and reducing clinical signs in more than half of patients was assessed [60]. However, confirmation of the results of the work required controlled clinical trial studies.

Common and standard treatments were performed for the control group in 11 clinical trial studies with HCQ. The results of these studies indicate that HCQ has no significant effect on reducing mortality and morbidity and improving clinical symptoms caused by COVID-19 [37,49,50,53,55,57,58,62,63]. In another work, the effect of HCQ in preventing the treatment of COVID-19 was measured, which also stated that HCQ is not effective in preventing the disease [27]. This result was also observed in the study of Mitjà O. et al. [49], in which, the authors examined healthy individuals exposed to COVID-19. Only in one work was the relative effect of HCQ on the recovery of patients with COVID-19 observed [51].

A comparison between HCQ and a placebo group was observed in four studies. Most of these studies have shown that HCQ has no effect on the recovery, reduction in symptoms, and prevention of mortality in patients with COVID-19 [29,35,36]. However, the results of one study indicate that the administration of the HCQ drug prevents the onset of severe symptoms of the disease and, in these patients, only mild gastrointestinal symptoms were observed [61].

In many other studies, HCQ was prescribed as a control drug in both groups. In the study of Ansarin et al., the effect of bromhexine with HCQ was measured. The results of this study showed that oral bromhexine has a positive effect on reducing mortality, transfer to the ICU, and the rate of patients’ intubation [38]. In another study, the effect of azithromycin with HCQ was measured in patients, but no positive results were obtained in this study [55]. Another article reported the examination of the effect of arbidol with HCQ in COVID-19 patients. This study stated that arbidol has a positive effect on reducing clinical symptoms, the patient respiratory status, ICU transfer, and hospitalization time [42]. However, it is not possible to confirm the results of these studies until wider trials are implemented.

The effect of azithromycin was evaluated in two studies. In one study, a significant relationship was observed between the improvement in patients‘ clinical symptoms [56] and, in another study, the positive effect of azithromycin on the improvement in patients’ symptoms was confirmed [45]. Two other studies measured the effect of interferon on COVID-19 patients. The results of these studies showed a reduction in mortality and clinical symptoms in patients with COVID-19 [33,39]. In four other studies performed in Iran, the effect of sofosbuvir in patients was measured. The results of three studies showed a positive effect of drug and recovery and reduction in patient mortality [40,41,44], while another study did not report any significant relationship between sofosbuvir use and patients’ recovery [42]. According to the information in Table 1, other drugs were tested in different trials, though, due to the limited population, confirmation of their results is subject to the implementation of larger trials in patients. The information of these studies is fully listed in Table 2.

### 3.2. Artificial Intelligence

We initialized the associations by selecting drugs where their *p*-values with COVID-19 is small. The sources of the selection of drugs that seems to be effective in treating COVID-19 are articles, clinical expert reports, and medical databases. Selected drugs include: ‘Baricitinib’ [66,67], ‘Tocilizumab’ [68,69], ‘Immucillin A’ [70], ‘Favipiravir’ [71], ‘Remdesivir’ [72], ‘Hydroxychloroquine’ [73], ‘Ivermectin’ [74], ‘Azithromycin’ [74,75], ‘BNT162′ ‘BBV152′, ‘Bamlanivimab’, ‘AZD1222′, ‘Arbidol’, ‘Ad5-nCoV’, ‘Ad26.COV2.S’, ‘Adenosine Monophosphate’, ‘Gam-COVID-Va’,’ ‘Etesevimab’, ‘Coro-naVac’, ‘Ciclesonide’, ‘Casirivimab’, ‘Camostat’, ‘Nafamostat’, ‘Moclobemide’, ‘Lopinavir’, ‘Lenzilumab’, ‘Ivermectin’, ‘Itolizumab’, ‘Interleukin 1 Receptor Antagonist Protein’, ‘INO-4800′, ‘Immucillin A’, ‘Therapeutic Corticosteroid’, ‘Siltuximab’, ‘Sarilumab’, ‘Ruxolitinib’, ‘Ritonavir’, ‘Regdanvimab’, ‘Recombinant Interferon Alpha 2b-like Protein’, ‘Oseltamivir’, ‘NVX-CoV2373′, and ‘Nitazoxanide’.

The p-NA algorithm calculates combined *p*-values between each of these drugs and COVID-19. Then, it selects the drug with the lowest combined *p*-value. After selecting the first drug, the p-NA updates weights between COVID-19 and interface-affected human genes according to the *p*- values between those genes and the selected drug. Thus, all of the combined *p*-values between these drugs and COVID-19 are updated. The p-NA iterates to find the drug with the lowest combined *p*-value by updating weights. At the end, the p-NA algorithm proposed scenarios, where each scenario contains a drugs recipe, as is the aim of p-NA for making associations cooperate. Table 3 and Table 4 show different scenarios proposed by p-NA for the drugs recipe. In Table 3, the threshold for stopping the algorithm is 0.05, whereas 0.01 is the threshold of p-NA in Table 4. The p-NA iterates finding drugs until the combined *p*-value between each scenario and COVID-19 become less than the threshold. Table 3 shows the proposed scenarios of artificial intelligence in drug combinations at a significance level of 0.05. The first scenario of Table 3 contains two drugs proposed by p-NA, which are as follows:

- Therapeutic corticosteroid with a combined *p*-value of 0.07;
- Camostat with a combined *p*-value of 0.02, on the condition that the therapeutic corticosteroid is selected in this scenario.

**Table 3:** Scenarios for drugs recipe with their combined *p*-values with threshold of 0.05.

**‘Therapeutic Corticosteroid’ (0.07234703) + ‘Camostat’ (0.024973529)**
‘Remdesivir’(0.125641) + ‘Azithromycin’ (0.032386)
‘Interleukin 1 Receptor Antagonist Protein’ (0.162201) + ‘Camostat’ (0.026755)
‘Chloroquine’ (0.172638) + ‘Favipiravir’ (0.083871) + ‘Camostat’ (0.046428)
‘Hydroxychloroquine’ (0.183845) + ‘Azithromycin’ (0.07669) + ‘Camostat’ (0.044154)

**Table 4:** Scenarios for drugs recipe with their combined *p*-values with threshold of 0.01.

**‘Interleukin 1 Receptor Antagonist Protein’ (0.162201) + ‘Camostat’ (0.026755) + ‘Azithromycin’ (0.010872) + ‘Tocilizumab’ (0.008609) + ‘Oseltamivir’ (0.006937)**
‘Interleukin 1 Receptor Antagonist Protein’(0.162201) + ’Camostat’(0.026755) + ’Chloroquine’(0.014835) + ’Favipiravir’ (0.00952)+ ’Tocilizumab’(0.007638)
‘Therapeutic Corticosteroid’ (0.07234703) + ‘Camostat’ (0.024973529) + ‘Oseltamivir’ (0.017227) + ‘Remdesivir’ (0.011593) + ‘Tocilizumab’(0.009214)

Therefore, the combined *p*-value between the drugs recipe in scenario 1 and COVID-19 is 0.02.

In other words, if only ‘Therapeutic Corticosteroid’ is used, the significance level is 0.07, and if this drug is combined with ‘Camostat’ and taken at the same time, the level is significantly reduced to 0.02 and has a better effect on the treatment of COVID-19 patients. The second scenario of Table 3 states that if only ‘Remdesivir’ is used to treat patients, the significance level is 0.1 and, if it is used concomitantly with ‘Azithromycin’, the significance level is reduced to 0.05, which again has a better effect than from taking ‘Remdesivir’ alone. Also, in the third scenario of Table 3, if the ‘Interleukin 1 Receptor Antagonist Protein’ is used for patients, the level of significance is 0.1 and, if this drug is taken simultaneously with the drug ‘Camostat’, the level of significance to it reaches 0.02. The fourth scenario in this table suggests a combination of three different drugs so that the use of ‘Chloroquine’ alone has a significance level of 0.17 and, if ‘Favipiravir’ is taken simultaneously with this drug, the significance level is 0.08 and, if ‘Camostat’ should be added to the combination of the other two drugs, the significance level reaches 0.046, which is a much more effective combination than the use of ‘Chloroquine’ alone for the treatment of patients with COVID-19. Finally, the fifth scenario in Table 3 shows the combination of the three drugs hydroxychloroquine, azithromycin, and camostat, in which, the AI predicts a *p*-value of this combination with COVID-19 of 0.044.

Based on the results of Table 4, in the combination of drugs for the treatment of patients with COVID-19, the proposed scenarios for the treatment of patients with COVID-19 at a significance level of 0.01 are as follows. In the first scenario of Table 4, when only interleukin 1 receptor antagonist protein is used to treat patients, the significance level is 0.16 and, if used concomitantly with camostat, the significance level is 0.02 and, if azithromycin is added, the significance level is 0.01 and, with the addition of tocilizumab and oseltamivir, a significant level is reached at 0.008 and 0.006, respectively, which is a very effective drug combination in the treatment of patients with COVID-19. Also, in the second scenario of Table 4, if interleukin 1 receptor antagonist protein is used alone, the significance level is 0.16, if camostat is added, the significance level is 0.02, if chloroquine is added, the significance level is 0.01, and, if two drugs, favipiravir and tocilizumab, are used, they reach significant levels of 0.009 and 0.007, respectively, making this combination an effective treatment. Finally, the third scenario of Table 4 shows that, in the case of taking therapeutic corticosteroid alone, a significant level of 0.07 is reached, with the addition of camostat, a significant level of 0.02 is reached, with the addition of oseltamivir, a significant level of 0.017 is reached, and with the addition of remdesivir and tocilizumab, the significance reaches 0.011 and 0.009, respectively, where another effective drug combination is suggested.

The *p*-values between affected human genes and COVID-19 are shown in Table 5. The second column shows the *p*-value between COVID-19 and the corresponding affected human genes in the first column of this table. The column with title “Scenario 1” demonstrated the combined *p*-value between COVID-19 and the drugs recipe in scenario 1 from 2. The combined *p*-values of other scenarios in 2 are shown as columns in 4. In each scenario of p-NA, the *p*-values between affected human genes and COVID-19 become close to 1. However, there are some genes in Table 4 where their *p*-values are still far from 1.

As was known until writing this paper, there is not much information in network meta-analyses for drugs that have small *p*-values with them.

**Figure 7:**
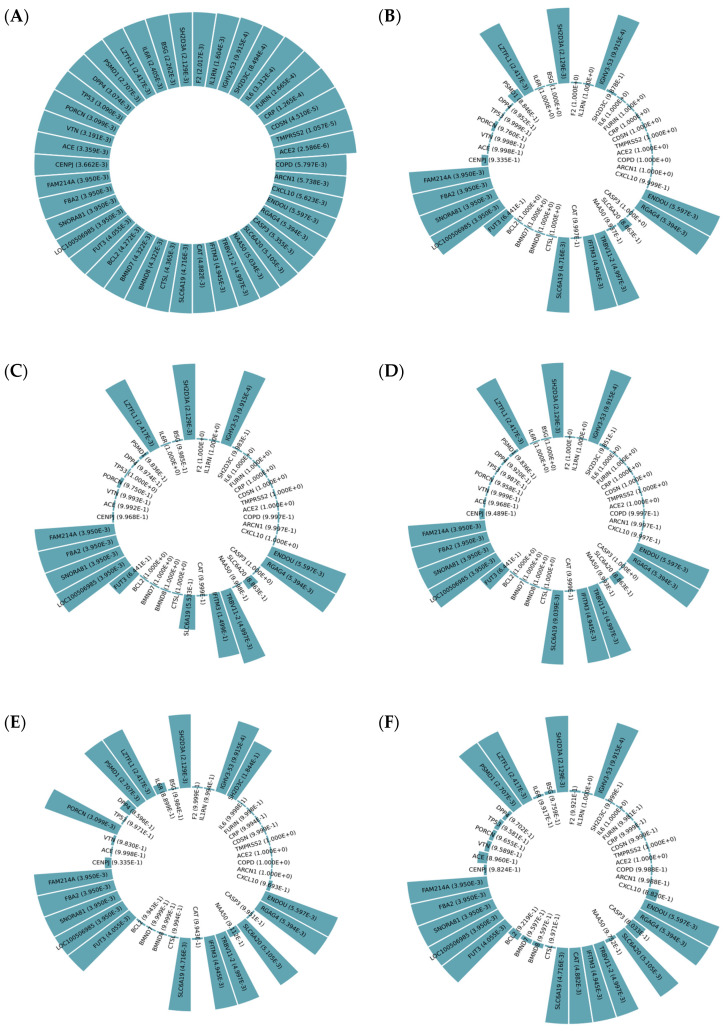
(**A**) The circular bar plot of Table 5 *p*-value column, (**B**) the circular bar plot of scenario 2 column, (**C**) the circular bar plot of scenario 1 column, (**D**) the circular bar plot of scenario 3, (**E**) the circular bar plot of scenario 4, (**F**) the circular bar plot of scenario 5.

**Figure 8:**
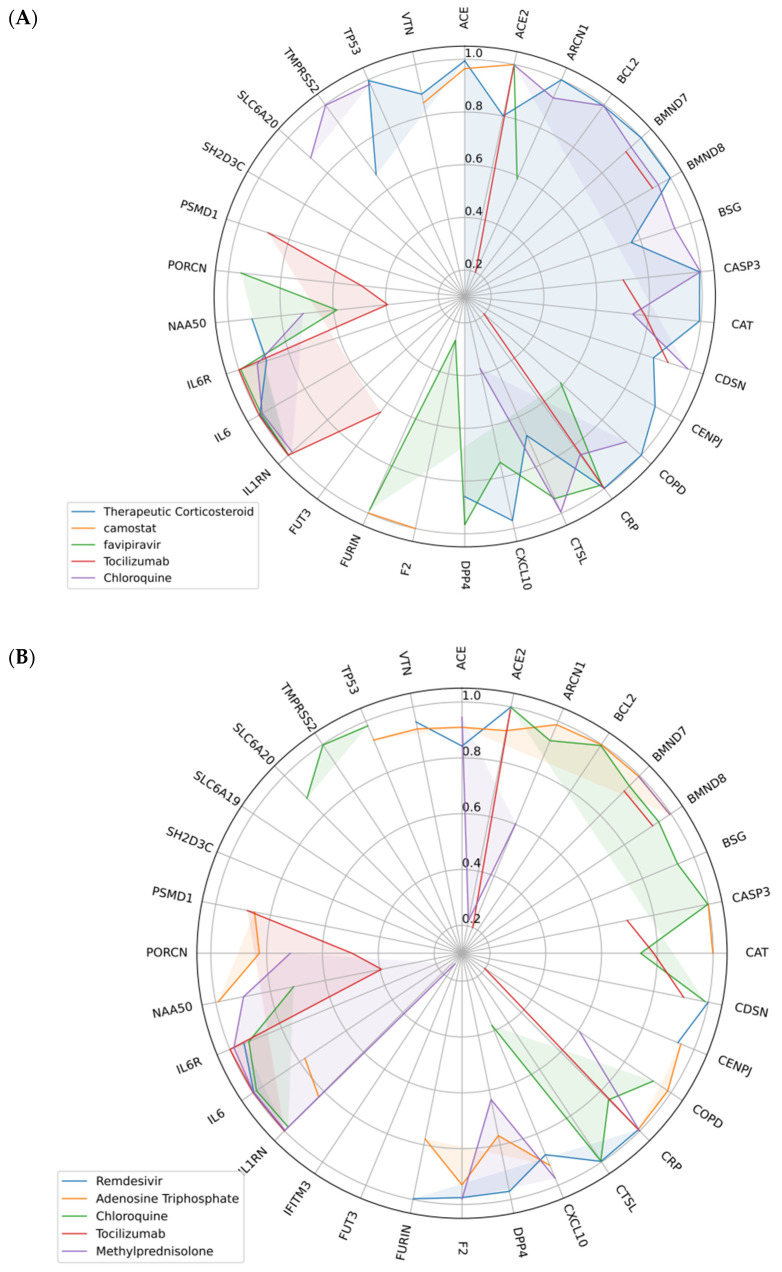

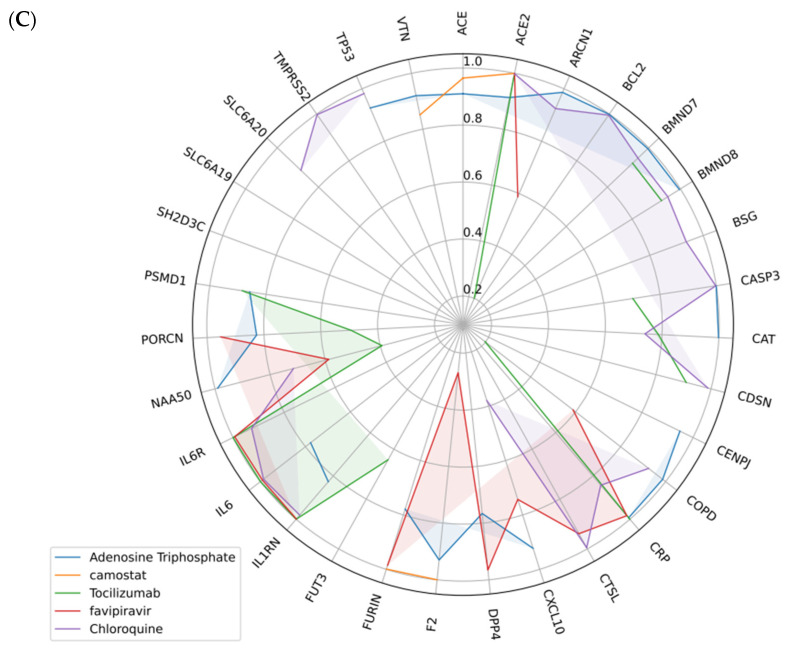
(**A**) The radar chart of scenario 1, (**B**) the radar chart of scenario 2, (**C**) the radar chart of scenario 3.

Whereas Figure 7A shows the *p*-values between affected human genes and COVID-19, Figure 7B–F demonstrate the *p*-values between them after implementing scenarios 1 to 5, respectively. In other words, Figure 7B–F show different circular bar plots to show the efficiencies of drugs selected using p-NA by demonstrating the *p*-values between COVID-19 and human genes after the consumption of selected drugs. Figure 8A–C, on the other hand, indicate *p*-values between affected human genes and COVID-19 after implementing three new scenarios. Each colored line shows the effectiveness of the corresponding drug in that scenario in raising the *p*-value.

**Figure 9:**
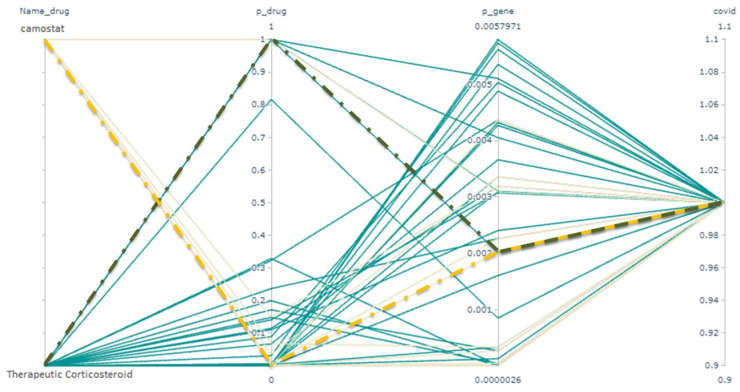
Network chart for *p*-values between COVID-19 and two drugs, with human genes as interface features.

Figure 9 shows the network chart for *p*-values between human genes and two different drugs, namely ‘Therapeutic Corticosteroid’ and ‘Camostat’. Their *p*-values with affected human genes are completely different. The network chart shows how two supplement drugs help each other to overcome COVID-19. For example, the dashed green line in this figure shows that the *p*-value between one of the human genes and COVID-19 is about 0.002. The *p*-value between that human gene and ’Therapeutic Corticosteroid’ is approximately one (green dashed lines), whereas the *p*-value between that human gene and ‘Camostat’ is close to zero (yellow dashed lines).

## 4. Discussion

COVID-19 is known to be a challenging disease for healthcare systems globally. The disease spreads rapidly within the community and, during a severe clinical course, leads to fatality among a considerable proportion of patients. Since there is no definitive cure for the disease, various treatment strategies were studied in the study, most of which were based on the use of antiviral drugs. However, factors such as different doses of drugs used, the interval between the onset of symptoms and the start of treatment, the severity of the disease in different patients enrolled in different trials, the lack of standard controls, and the lack of controlled randomized trials due to long contradictory results, were reported in previous studies [76].

One of the methods that can be used to solve the problems caused by heterogeneity and limitations of studies is the use of artificial intelligence techniques. Using advanced machine learning algorithms, the integration and analysis of data related to COVID-19 is conducted with higher speed and accuracy. Accordingly, it is possible to develop effective treatments and identify new treatment approaches for COVID-19 [11].

In this article, using WGM, we implemented the proposed p-NA method for COVID-19 treatment. In this section we, at first, focus on the advantages of WGM over the previously known combining *p*-values method. Second, we discuss the drawbacks of p-NA. Then, we discuss alternatives for associations in treating COVID-19.

In the present study, the results of 39 interventional studies were reviewed. Our studies showed a high heterogeneity for the effectiveness of different drugs. Evidence suggests that hydroxychloroquine’s efficacy in reducing the symptoms and mortality of COVID-19 patients is weak, and cannot be described as an effective treatment for COVID-19 patients. Our findings are consistent with the results of previous studies, so that, in the study of Thibault et al. [77], which was conducted as a systematic review and meta-analysis, it was observed that HCQ alone cannot be effective in treating patients with COVID-19. In addition, this study showed that the simultaneous use of HCQ and azithromycin in the treatment of patients with COVID-19 increases the mortality caused by this disease. Another cohort study in the United States found that the use of HCQ, azithromycin, or both had no positive effect on improving patients and reducing their hospital mortality [78]. However, the results of our study showed that the simultaneous use of these two drugs is more effective than the use of HCQ or azithromycin alone.

Other drugs, including tocilizumab, were also used to treat patients with COVID-19. Previously, some open-label, non-randomized studies have shown that blocking early interleukin-6 receptors helps to reduce the severity of COVID-19. The results of limited RCT studies in this field indicate that this drug alone cannot be considered an effective treatment for COVID-19 [52,79].

The scenarios proposed by artificial intelligence in this study show that the addition of tocilizumab to various drug combinations significantly increases the effectiveness of treatments. This is also true for other drugs, including remdesivir. Thus, previous studies examining the effectiveness of remedial in patients with COVID-19 showed that this drug alone could not be recognized as an appropriate treatment for COVID-19 [31].

Interferon beta, lopinavir-ritonavir, sofosbuvir, calcifediol, and colchicine were other drugs used to treat COVID-19. Despite the positive efficacy of these drugs in studies, since the standard clinical trials with a high number of participants have not been performed, the effectiveness of these drugs cannot be considered with certainty. However, the evidence from artificial intelligence in our study shows that the combination of these drugs can have far better effects.

### 4.1. Advantages of WGM in Combining p-Values

Fisher’s test is not the best test available to combine *p*-values of multiple tests of the same null hypothesis [80]. Fisher’s method does not use weights, while different weights can be assigned to each study according to their power [21,81]. In our study, 1 − *p*-values were viewed as weights of affected human genes. Stouffer’s method uses the Z-transform to find the combined *p*-values. One generalized version uses the weights to each study. However, all of them assume that all of the null hypotheses are true. The Z-transform of 0 and 1 are negative and positive infinity, respectively. The geometric mean is one of the correct means when averaging normalized results to [0,1] [82], in which, *p*-values satisfy the condition.

### 4.2. Drawbacks of the p-NA

Network meta-analysis computes the *p*- values between two associations. However, the effectiveness between two associations is not mentioned. For instance, the p-values between COVID-19 and both remdesivir and recombinant interleukin-6 are small in network meta-analysis. However, recombinant interleukin-6 should be decreased for patients with COVID-19. Remdesivir, on the other hand, is a good drug in treating COVID-19. Greedy algorithms find the locally optimal choice, where the solution is found. In most cases, the result is not the optimal solution. For example, in the travelling salesman problem, the city with the shortest distance is always selected, while the selected tour may be the worst tour [75].

### 4.3. Alternatives for Associations

Sequence similarity searching is a method of searching sequence databases by using alignment to a query sequence. By statistically assessing how well the database and query sequences match, one can infer homology and transfer information to the query sequence. The tools can be launched with different form presets using the links. These can be changed on the tool page as well.

Sequence similarity searching is a method of searching for sequence databases by aligning them with a query sequence. By statistically evaluating how closely the database and query sequences match, we can derive homology and transfer information to the query sequence. The tools can be started with various form presets via links. Related diseases: in recent years, there have been three types of coronaviruses: severe acute respiratory syndrome (SARS-CoV), Middle East respiratory syndrome (MERS), and coronavirus disease 2019 (COVID-19). The first one, SARS-CoV, spread over months in 2002 and has symptoms that include a high fever, headache, body aches, and a dry cough. The disease is transmitted through the mouth, nose, or eyes, such as kissing, touching, sharing utensils for eating and drinking, or talking with an infected person. There is no treatment for SARS. Scientists are testing treatments and vaccines. The second disease, MERS, was first reported in 2012 in a pneumonia patient. Symptoms include renal failure and severe acute pneumonia, with often fatal outcomes. Many people with the third one, COVID-19, also have pneumonia-viral infection in lungs. Chest x-rays and blood tests can help to determine the infection. However, preventing pneumonia is always better than treating it. Symptoms: COVID-19 has symptoms such as fever, cough, and shortness of breath (dyspnea). However, other coronaviruses have a sore throat and headache as their other symptoms.

## 5. Conclusions

We have proposed a new machine learning algorithm, namely p-NA, to find a combinations of drugs (associations) for COVID-19 treatment. The drugs that are examined in p-NA are the ones where the *p*-values between them and COVID-19 are smallest in the network meta-analysis. The p-NA, on the other hand, uses interface features (human affected genes in this work) to compute the *p*-values between the target COVID-19 and associations (drugs). The *p*-values between drugs and these human genes, and the weights (1 − *p*-values) between human genes and COVID-19, lead to computing a combined *p*-value between drugs and COVID-19. Since *p*-values follow the uniform distribution, the combination method is calculated by a weighted geometric mean. Sorting in ascending order of the combined *p*-values between COVID-19 and drugs, p-NA selects the first drug. After selecting this drug, the weights between human genes and COVID-19 are updated; consequently, the combined *p*-values are updated. The process of selecting the drug with the smallest combined *p*-value and updating the combined *p*-value iterates until the p-NA becomes less than the threshold. Two different thresholds in this work are suggested as 0.05 and 0.01, i.e., the combined *p*-values between COVID-19 and drugs. The p-NA implemented with both thresholds and different scenarios of drug combinations are proposed. The results show that the proposed combined drugs resulted in a *p*-value close to 1 between COVID-19 and important affected human genes (such as FURIN, ACE2, etc.).

## Figures and Tables

**Table 1:** Similarity measures between proteins of COVID-19 and nucleotides.

Nucleotide	Count	Mean per Ident	std per Ident	Mean E-Value	std E-Value	Mean Query Cover	std Query Cover
Pneumonia viral	163	94.99	11.8	3.99 × 10^−2^	2.24 × 10^−1^	99.76	1.36
Pangolin coronavirus isolate MP789	22	91.19	11.95	7.27 × 10^−2^	3.33 × 10^−1^	99.91	0.29
SARS-CoV	2518	90.06	12.78	2.12 × 10^−2^	1.65 × 10^−1^	99.58	1.62
Betacoronaviruses	815	80.34	12.91	7.36 × 10^−8^	1.56 × 10^−6^	99.63	1.59
SARS-like coronavirus WIV16	17	75.08	9.001	3.53 × 10^−22^	9.67 × 10^−22^	99.76	0.64
Rhinolophus affinis coronavirus	12	73.27	9.777	3.33 × 10^−12^	1	1.11 × 10^−11^	99.92

**Table 2:** Demographic information.

First Author, Year, (Ref)	Site	Intervention Group (Participants, Mean Age, Male%, Drug)	Procedure Intervention Group	Control Group/Placebo (Participants, Mean Age (SD), Male%, Drug)	Procedure Control Group	Parameters	Comments
Abd-Elsalam, S. 2020, [62]	Egypt	97 patients with COVID-19, 40.35 ± 18.65, HCQ+ standard care	HCQ 400 mg twice daily (in day 1) then HCQ 200 mg twice daily (in day 2–15) with standard care treatment by the Egyptian MOH for 15 days	97 patients with COVID–19, 41.09 ± 20.07, Standard care	Standard care treatment by the Egyptian MOH for 15 days	Risk of mortality in HCQ treatment: OR = 0.824, *p* = 0.725	HCQ treatment was not significantly associated with reduced mortality in COVID-19 patients
Abella, B.S. 2021, [27]	America	66 HCWs with the aim of the prevention, 31 (20–66), HCQ	HCQ 200 mg tablets in 3 times a day with food (8 week)	66 HCWs	Placebo group received custom-molded identically sized and shaped microcrystalline cellulose tablets	N/A	HCQ had no advantage in preventing COVID-19 in HCWs
Annweiler, C. 2020, [65]	France	57 residents with COVID-19 from nursing-home, 87.7 ± 9.3, 21% Vitamin D supplement	Take a vitamin D3 supplement during COVID-19 or in the month before	9 residents with COVID-19 from nursing-home, 87.4 ± 7.2, 33%	N/A	Risk of mortality: HR = 0.11, *p* = 0.003 OSCI score: Β = −3.84, *p* = 0.001	Vitamin D3 supplement bolus reduced the severity of the disease and increased the survival of the elderly
Ansarin, K. 2020, [38]	Iran	39 patients in ICU, 58.4 ± 13.7, 48.8%, Bromhexine hydrochloride and standard care	oral bromhexine hydrochloride 8 mg three times a day and standard care (Hydroxychloroquine 200 mg/d for two weeks) for two weeks	39 patients in ICU, 61.1 ± 16.1, 61.5%, Standard care	Hydroxychloroquine 200 mg/d for two weeks	ICU admissions: 2 out of 39 intervention V.S 11 out of 39 control, *p* = 0.006 Intubation 1 out of 39 intervention vs. 9 out of 39 control, *p* = 0.007 Death 0 in intervention vs. 5 in control, *p* = 0.027	Timely administration of oral bromhexine reduced mortality, reduced transfer to ICU, reduced intubation
Ayerbe, L. 2020, [46]	Spain	1734 COVID-19 patients, 68.77 ± 15.09, 60.4%, Heparin	Heparin in 2–15 days	N/A	N/A	Adjusted OR for age and gender = 0.55, *p* = 0.003, OR oxygen < 90%, and temperature > 37 °C = 0.54, *p* = 0.003 Other drugs were included as covariates OR = 0.42, *p* < 0.001	Heparin appeared to be involved in reducing mortality. However, randomized controlled trials are required for further investigation.
Ayerbe, L. 2020, [47]	Spain	1857 patients with COVID-19, 67.11 ± 15.51, 62.04%, HCQ	HCQ in 2–15 days	N/A	N/A	Adjusted for age and gender, with OR = 0.44, *p* < 0.001, OR oxygen < 90%, and temperature > 37 °C = 0.45, *p* = < 0.001	HCQ appeared to be involved in reducing mortality. However, randomized controlled trials are required for further investigation
Barnabas, R.V. 2021, [28]	America	353 40 (27–51), 39%, HCQ	HCQ (400 mg/d for 3 days followed by 200 mg/d for 11 days)	336, 38 (26–50), 41% Ascorbic acid	Ascorbic acid (500 mg/d followed by 250 mg/d)	HR SARS-CoV-2 acquisition = 1.10, *p* > 0.20 Frequency of participants experiencing adverse events: 66 in HCQ group vs. 46 in control group, *p* = 0.026	The results of the study indicate a significant effect of HCQ as prevention after exposure to the prevention of SARS-CoV-2 infection.
Boulware, D.R. 2020, [29]	America	414, 41 (33–51), 47.3%, HCQ	HCQ (800 mg once, followed by 600 mg in 6 to 8 h, then 600 mg daily for 4 additional days)	407, 40 (32–50), 49.4%	Placebo folate tablets, which were similar in appearance to the HCQ	The incidence of new illness: 11.8% in HCQ group vs. 14.4% in placebo group	The incidence of new illness compatible with COVID-19 did not differ significantly between groups
Brown, S.M. 2021, [30]	America	42, 51 (42–60), 54% HCQ	HCQ, drug was held if QTc > 500 msec	43, 58 (43–68), 67, Azithromycin	Azithromycin was held if QTc > 500 msec	_	Studies have shown that neither drug is superior to the other. It was also observed that renal complications of HCQ were greater than azithromycin, which may be due to chance
Cavalcanti, A.B. 2020, [55]	Brazil	217, 49.6 ± 14.2, 56.7%, HCQ plus plus azithromycin, 221, 51.3 ± 14.5, 64.3%, HCQ	Standard care plus HCQ at a dose of 400 mg twice daily plus azithromycin at a dose of 500 mg once daily for 7 days standard care plus HCQ at a dose of 400 mg twice daily	227, 49.9 ± 15.1, 54.2%, Standard care	Standard care	OR (HCQ alone): 1.21, *p* = 1 OR (HCQ plus azithromycin) = 0.99, *p* = 1	The use of HCQ alone or with azithromycin did not improve clinical status at 15 days compared with standard care
Chen, C.P. 2020 [63]	Taiwan	21, 33 ± 12 52.4%, HCQ	HCQ (400 mg twice for 1 d or HCQ 200 mg twice daily for 6 days) plus standard care	12, 32.8 ± 8.3, 66.7	Standard care		Neither study demonstrated that HCQ shortened viral shedding in mild to moderate COVID-19 subjects.
Davoudi-Monfared, E. 2020 [39]	Iran	42, 56.50, 52.4%, IFN	44 micrograms/mL (12 million IU/mL) of interferon β-1a was subcutaneously injected three times weekly for two consecutive weeks	39, 61.00, 56.4, Control	National protocol medications	Overall mortality: OR = 2.5, *p* < 0.05, Early administration reduced mortality: OR = 13.5	The use of IFN had a significant effect on reducing mortality
Deftereos, S.G. 2020, [64]	Greek	55, 63, 56.4%, Colchicine	1.5 mg loading dose followed by 0.5 mg after 60 min and maintenance doses of 0.5 mg twice daily plus standard care	50, 65, 60	Standard care	Clinical deterioration control vs. intervention: OR = 0.11, *p* = 0.02	Participants who received colchicine had statistically significantly improved time to clinical deterioration
Dequin, P.F. 2020, [50]	France	76, 63.1 (51.5–70.8), 71.1%, HCQ	200 mg/d until day 7 and then decreased to 100 mg/d for 4 days and 50 mg/d for 3 days, for a total of 14 days	73, 66.3 (53.5–72.7), 68.5, Placebo	Standard care	_	Low-dose hydrocortisone, compared with placebo, did not significantly reduce treatment failure
Entrenas Castillo, M. 2020, [48]	Spain	50, 53.14 ± 10.77, 54% Calcifediol	(400 mg every 12 h on the first day, and 200 mg every 12 h for the following 5 days), azithromycin (500 mg orally for 5 days PLUS oral calcifediol (0.532 mg)	26, 52.77 ± 9.35, 69%, Control	(400 mg every 12 h on the first day, and 200 mg every 12 h for the following 5 days), azithromycin (500 mg orally for 5 days	Multivariate risk estimate odds ratio for ICU: intervention vs. control OR = 0.02	Calcifediol in combination with standard treatment significantly reduced the need for ICU treatment in patients requiring hospitalization due to COVID-19
Eslami, G. 2020, [40]	Iran	35, 62 (47–69), 49% sofosbuvir/daclatasvir	One arm received a single daily pill containing 400 mg sofosbuvir and 60 mg daclatasvir	27, 60 (43–73), 52, Ribavirin	600 mg ribavirin every 12 h	relative risk of death for patients treated with sofosbuvir/daclatasvir: RR = 0.17, *p* = 0.02 relative risk of number needed to treat for benefit: RR: 3.6, *p* < 0.01	Treatment of patients with severe COVID-19 with sofosbuvir/daclatasvir was significantly more effective than ribavirin through improved clinical symptoms
Furtado, R.H.M. 2020, [56]	Brazil	214, 59.4 (49.3–70.0), 60%, Azithromycin	Azithromycin (500 mg via oral, nasogastric, or intravenous administration once daily for 10 days) plus standard of care (HCQ 400 mg twice daily for 10 days)	183, 60.2 (52.0–70.1) 67%, Control	Standard of care (HCQ 400 mg twice daily for 10 days)	OR = 1.36, *p* = 0.11	There was no significant difference in terms of side effects and effective treatment between the two groups
Garibaldi, B.T. 2021, [31]	America	342, 60 (46–69), 55.3%, remdesivir	Remdesivir plus corticosteroid administration.	1957, 60 (44–74), 51.3%, Control	Without remidisivir treatment	Clinical improvement: HR = 1.47, Mortality: HR = 0.7 *p* > 0.05	The differences between the two groups were not significant in terms of mortality reduction and recovery rate
Gautret, P. 2020, [51]	France	20, 51.2 ± 18.7, 45%, HCQ	Hydroxychloroquine sulfate 200 mg, three times per day for ten days	16, 37.3 ± 24.0, 37.5%, Control	Standard care	_	Twenty cases were treated in this study and showed a significant reduction in the viral carriage at D6 post-inclusion compared to controls
Hermine, O. 2021, [52]	France	63, 64.0 (57.1–74.3), 70%, Tocilizumab	8mg/kg, intravenously plus usual care on day 1 and on day 3 if clinically indicated	67, 63.3 (57.1–72.3), 66%,	Usual care	Noninvasive ventilation (NIV) or mechanical ventilation (MV) or more died in the TCZ group than in the UC group: HR = 0.58,	May reduce the risk of NIV, MV, and death. Further studies are needed to confirm these preliminary results.
Horby, P. 2020, [53]	England	1561, 65.2 ± 15.2, 61.5%, HCQ	A 200 mg tablet containing a 155 mg base equivalent) in a loading dose of four tablets (total dose, 800 mg) at baseline and at 6 h, which was followed by two tablets (total dose, 400 mg) starting at 12 h after the initial dose and then every 12 h for the next 9 days	3155, 65.4 ± 15.4, 62.6%	Usual care	Death within 28 days: RR = 1.09. *p* = 0.15 discharged from the hospital alive within 28: RR = 0.9, invasive mechanical ventilation or death: RR: 1.14	Among patients hospitalized with COVID-19, those who received HCQ did not have a lower incidence of death at 28 days than those who received usual care
Horby, P.W. 2020, [54]	England	1616, 66·0 ± 16·0, 60%, Lopinavir–ritonavir	(400 mg and 100 mg, respectively) by mouth for 10 days	3424, 66·4 ± 15·8, 61%	Usual care	died within 28: RR = 1.03, *p* = 0.6. Discharged from hospital alive within 28 days: RR = 0.98, *p* = 0.53 invasive mechanical ventilation or death: RR = 1.09, *p* = 0.092	In patients admitted to hospital with COVID-19, lopinavir–ritonavir was not associated with reductions in 28-day mortality, duration of hospital stay, or risk of progressing to invasive mechanical ventilation or death
Kasgari, H.A. 2020, [41]	Iran	24, 45 (38–69), 46%, sofosbuvir/daclatasvir/ribavirin	400 mg sofosbuvir, 60 mg daclatasvir, and 1200 mg ribavirin	24, 60 (47.5–68.5), 29%	Standard care	Number of ICU admission: intervention vs. control (0 versus 4, *p* = 0.109) Number of deaths: intervention vs. control (0 versus 3, *p* = 0.234)	The number of ICU admissions in the sofosbuvir/daclatasvir/ribavirin group was not significantly lower than the control group There was no difference in the number of deaths between the groups
Langer-Gould, A. 2020, [32]	America	52, 59.8 ± 11.7, 86% Tocilizumab	1–4 doses of tocilizumab median of 14 days	41, 58.8 (12.7), 68.3%, Anakinra	Anakinra, a median of 14 days	_	_
Lofgren, S.M. 2020, [61]	Canada	658, _ _ HCQ	800 mg load dosing, followed by 600 mg 6–8 h later, and then 600 mg daily for 5 days in total.	654, _ _ Placebo	_		Data from 3 outpatient COVID-19 trials demonstrated that gastrointestinal side effects were common but mild with the use of hydroxychloroquine, whereas serious side effects were rare
Lyngbakken, M. N. 2020, [57]	Norway	27, 56 (41, 72), 70.4% HCQ	At a dose of 400 mg twice daily for 7 days)	26, 69 (51, 74), 61.5%	Standard care	Died in hospital: OR = 1.11, *p* > 0.05	Therapy with hydroxychloroquine did not impact SARS-CoV-2 viral kinetics in patients admitted to hospital with moderately severe COVID-19.
Mitjà, O. 2021, [49]	Spain	1116, 48.6 ± 18.7, 27.3 HCQ	The drug at a dose of 800 mg once, followed by 400 mg daily for 6 days	1198, 27, 27%	Usual care	The incidence of PCR-confirmed symptomatic COVID-19: RR = 0.86, *p* > 0.05	Post-exposure-therapy with HCQ did not prevent SARS-CoV-2 infection or symptomatic COVID-19 in healthy persons exposed to a PCR-positive case patient
Nojomi, M. 2020, [42]	Iran	50, 56.6 ± 17.8, 66% Arbidol plus HCQ	HCQ (400 mg BD on first day) followed by ARB (200 mg TDS) 7 to 14 days	50, 56.6 ± 17.8, 54% Kaletra plus HCQ	HCQ (400 mg on first day) followed by 400 mg Kaletra (lopinavir/ritonavir) BD 7 to 14 days	_	Our findings showed that arbidol, compared to Kaletra, significantly contributes to clinical and laboratory improvements, including peripheral oxygen saturation, requiring ICU admissions, duration of hospitalization
Rahmani, H. 2020, [33]	America	33, 60 (47–73), 60.6% Interferon	IFN β-1b (250 mcg subcutaneously every other day for two consecutive weeks)	33, 61 (50–71), 57.5% Control	(lopinavir/ritonavir or atazanavir/ritonavir plus HCQ for 7–10 days)	Time to clinical improvement: HR= 2.30, *p* = 0.002. discharged patients OR = 3.09, *p* = 0.03 ICU admission rate: *p* = 0.04 All-cause 28-day mortality: 6.06% vs. 18.18%, *p* = 0.12	IFN β-1b was effective in shortening the time to clinical improvement without serious adverse events in patients with severe COVID-19. Furthermore, admission in ICU and need for invasive mechanical ventilation decreased following administration of IFN β-1b
Roozbeh, F. 2021, [43]	Iran	27, 43 (37–52), 44%, sofosbuvir/daclatasvir	Azithromycin capsules (500 mg for 6 days) with naproxen tablets (500 mg, twice daily for 7 days), as well as 40 mg pantoprazole tablets.,plus single daily oral tablet containing 400 mg sofosbuvir and 60 mg daclatasvir with HCQ (200 mg twice daily) for 7 days,	28, 47.5 (37–53), 50%	Azithromycin capsules (500 mg for 6 days) with naproxen tablets (500 mg, twice daily for 7 days), as well as 40 mg pantoprazole tablets.	_	Sofosbuvir/daclatasvir did not significantly alleviate symptoms after 7 days of treatment compared with control. Although fewer hospitalizations were observed in the sofosbuvir/daclatasvir arm, this was not statistically significant. Sofosbuvir/daclatasvir significantly reduced the number of patients with fatigue and dyspnea after 1 month
Sadeghi, A. 2020, [44]	Iran	33, 58 (38–65), 61% sofosbuvir/daclatasvir	HCQ 200 mg twice daily with or without lopinavir/ritonavir 200 mg/50 mg twice daily PLUS single daily oral tablet containing 400 mg sofosbuvir and 60 mg daclatasvir and standard care 14 days	33, 62 (49–70), 42% Control	HCQ 200 mg twice daily with or without lopinavir/ritonavir 200 mg/50 mg twice daily.	Clinical recovery within 14 days: 88% VS. 66%. *p* = 0.076, Median duration of hospitalization: 6 days vs. 8 days, *p* = 0.029. Incidence of hospital discharge: (Gray’s *p* = 0.041).	The addition of sofosbuvir and daclatasvir to standard care significantly reduced the duration of hospital stay compared with standard care alone. Although fewer deaths were observed in the treatment arm, this was not statistically significant.
Salvarani, C. 2021, [60]	Italy	60, 61.5 (51.5–73.5), 66.7%, Tocilizumab	Tocilizumab intravenously within 8 h from randomization at a dose of 8 mg/kg up to a maximum of 800 mg, followed by a second dose after 12 h.	66, 60.0 (54.0–69.0), 56.1%, Standard care	supportive care following the treatment protocols of each center	clinical worsening: RR: 1.05, died before 30: 2 vs. 1 patients Intubated: 6 vs. 5 patients	No benefit toward disease progression was observed compared with standard care.
Satlin, M.J. 2020, [34]	America	153, 62 (47–74), 63% HCQ	A dosage of 600 mg of HCQ every 12 h for two doses, followed by 400 mg daily for four additional days	N/A	N/A	-	HCQ appears to be reasonably safe and tolerable in most hospitalized patients with COVID-19. However, nearly one half of patients did not improve with this treatment
Sekhavati, E. 2020, [45]	Iran	56, 54.38 ± 15.92, 50%, Azithromycin	Oral AZM 500 mg daily, oral LPV/r 400/100 mg twice daily, and oral HCQ 400 mg daily (5 days)	55, 59.89 ± 15.55, 41.8%	Oral LPV/r 400/100 mg twice daily and oral HCQ 400 mg daily (5 days)	SpO 2 at discharge: Hedges g: −0.461, RR at discharge: 0.721 Length of hospital stay: 0.618	The SpO 2 levels at discharge were significantly higher, the respiratory rate was lower, and the duration of admission was shorter in the case group. There was no significant difference in the mortality rate between the two groups
Self, W.H. 2020, [35]	America	242, 58 (45–69), 55.8%, HCQ	400 mg of HCQ sulfate in pill form twice a day for the first 2 doses and then 200 mg in pill form twice a day for the subsequent 8 doses, for a total of 10 doses over 5 days	237, 57 (43–68), 55.7%	Matching placebo in the same dosing frequency. Patients discharged from the hospital before day 5 continued the trial medication after discharge to complete the 10-dose course	Clinical status: aOR = 1.02, *p* > 0.05, died: aOR = 1.07, *p* > 0.05	Among adults hospitalized with respiratory illness from COVID-19, treatment with HCQ compared with placebo did not significantly improve clinical status at day 14
Skipper, C. P. 2020, [36]	America	212, 41 (33–49), 42% HCQ	800 mg (4 tablets) once, then 600 mg (3 tablets) 6 to 8 h later, then 600 mg (3 tablets) once daily for 4 more days (5 days in total)	211, 39 (31–50), 45.5	Folic acid, 400 mcg, placebo tablets	difference in symptom severity: *p* = 0.117. Medication adverse effects: 43% VS. 24%, *p* = *p* < 0.001, Death: *p* = 0.29	HCQ did not substantially reduce symptom severity in outpatients with early, mild COVID-19.
Tang, W. 2020, [58]	China	75, 48.0 (14.1), 56% HCQ + standard care	1200 mg daily for three days followed by a maintenance dose of 800 mg daily. duration: two or three weeks	75, 44.1 (15.0), 53%	Standard care	_	HCQ did not result in a significantly higher probability of negative conversion than standard of care alone in patients admitted to hospital with mainly persistent mild to moderate COVID-19
Ulrich, R.J. 2020, [37]	America	67, 66.5 (16.4), 67.2% HCQ	HCQ 400 mg (2 tablets) by mouth two times per day (day 1) and 200 mg (1 tablet) by mouth two times per day (days 2–5); the five-day	61, 65.8 (16.0), 50.8%	Citrate was 400 mg (2 tablets) by mouth two times per day (day 1) and 200 mg (1 tablet) by mouth two times per day (days 2–5)	Severe disease progression endpoint: 11 people vs. 6, *p* = 0.350	HCQ did not prevent severe outcomes or improve clinical scores
Urwyler, P. 2020, [59]	Switzerland	5, 60 (54–81), 80%, Conestat alfa	Conestat alfa was administered by intravenous injections of 8400 IU followed by 3 additional doses of 4200 IU in 12-h intervals. Five patients	15, 59 (51–71), 80%	Control	_	Targeting multiple inflammatory cascades by conestat alfa was safe and associated with clinical improvements in the majority of severe COVID-19 patients

**Table 5:** *p*-values between human genes and COVID-19 before/after implementing the scenarios.

Human Gene	*p*-Value	Scenario 1	Scenario 2	Scenario 3	Scenario 4	Scenario 5
ACE2	2.58614 × 10^−6^	0.999965	0.99999	0.9999	0.9999	0.99999
TMPRSS2	1.05708 × 10^−5^	0.99999	0.99999	0.9999	0.9999	0.999988
CDSN	4.51014 × 10^−5^	0.9999	0.99993	0.999	0.9999	0.99965
CRP	0.0001264	0.9993	0.99994	0.9999	0.99741	0.99999
FURIN	0.000266511	0.99984	0.99814	0.9990	0.9999	0.96877
IL6	0.000331227	0.99978	0.99998	0.9999	0.9999	0.999985
SH2D3C	0.0008493	0.18437	0.99988	0.00084	0.98506	0.99689
IGHV3-53	0.000991476	0.00099	0.000991	0.00099	0.000991	0.000991
IL1RN	0.0016043	0.99938	0.99999	0.9999	0.99997	0.999994
F2	0.00201681	0.999930	0.992066	0.9999	0.9999	0.99998
SH2D3A	0.00212852	0.002128	0.002128	0.0021	0.002128	0.00212
BSG	0.00226155	0.99835	0.97591	0.99303	0.9999	0.99303
IL6R	0.00240459	0.889887	0.99172	0.99834	0.99951	0.998715
LZTFL1	0.00241652	0.00241	0.002416	0.002416	0.00241	0.002416
PSMD1	0.00270668	0.00270	0.00270	0.00270	0.00270	0.00270
DPP4	0.00307444	0.85959	0.97018	0.98462	0.96566	0.55886
TP53	0.00308951	0.997120	0.95810	0.62219	0.98204	0.99089
PORCN	0.00309865	0.003098	0.965501	0.00309	0.952501	0.99323
VTN	0.00319142	0.98295	0.95893	0.92295	0.990806	0.97145
ACE	0.00335915	0.99980	0.89595	0.97707	0.96491	0.98255
CENPJ	0.00366157	0.93348	0.98239	0.00366	0.00366	0.90752
FAM214A	0.00395025	0.00395	0.00395	0.00395	0.003950	0.003950
F8A2	0.00395025	0.00395	0.00395	0.00395	0.003950	0.003950
SNORA81	0.00395025	0.0039502	0.00395	0.00395	0.003950	0.003950
LOC100506985	0.00395025	0.003950	0.00395	0.00395	0.003950	0.003950
FUT3	0.00405499	0.004054	0.00405	0.0040	0.00405	0.00405
BCL2	0.0042722	0.99430	0.92189	0.02736	0.99526	0.92189
BMND7	0.00432166	0.99988	0.95967	0.94800	0.948063	0.97608
BMND8	0.00432166	0.99988	0.95967	0.94800	0.948063	0.97608
CTSL	0.00436471	0.99935	0.99705	0.99799	0.99999	0.99990
SLC6A19	0.00471616	0.00471	0.00471	0.00471	0.0047161621	0.004716
CAT	0.00488151	0.99434	0.00488	0.77965	0.7412232	0.90418
IFITM3	0.00494488	0.00494	0.004944	0.00494	0.00494488	0.00494
TRBV11-2	0.00499686	0.00499	0.004996	0.00499	0.004996	0.00499
NAA50	0.00503439	0.91120	0.97522	0.00503	0.883134	0.99118
SLC6A20	0.00510482	0.00510	0.00510	0.00510	0.88626	0.00510
CASP3	0.00535504	0.99511	0.80333	0.96211	0.998222	0.80333
RGAG4	0.00539409	0.00539	0.00539	0.00539	0.00539	0.00539
ENDOU	0.00559688	0.00559	0.00559	0.00559	0.005596	0.00559
CXCL10	0.00562288	0.96926	0.88201	0.99996	0.84129	0.97786
ARCN1	0.00573792	0.99997	0.99883	0.688791	0.96910	0.99926
COPD	0.00579706	0.99997	0.99883	0.69063	0.96913	0.99926

## Data Availability

Not applicable.
